# A Case of Erythropoietin (EPO)-Induced Pure Red Cell Aplasia and Its Treatment Efficacy With Desidustat

**DOI:** 10.7759/cureus.62022

**Published:** 2024-06-09

**Authors:** Pranjal Kashiv, Sunny Malde, Shubham Dubey, Sushrut Gupta, Twinkle Pawar, Kapil N Sejpal, Prasad Gurjar, Amit Pasari, Manish Balwani, Amol Bhawane, Priyanka Tolani, Charulata P Bawankule

**Affiliations:** 1 Nephrology, Jawaharlal Nehru Medical College, Wardha, IND; 2 Medicine, Jawaharlal Nehru Medical College, Datta Meghe Institute of Medical Sciences (Deemed to be University), Wardha, IND; 3 Nephrology, Saraswati Kidney Care Center, Nagpur, IND; 4 Nephrology, All India Institute of Medical Sciences, Nagpur, Nagpur, IND; 5 Internal Medicine, Jawaharlal Nehru Medical College, Wardha, IND

**Keywords:** erythropoetin, anemia, bone marrow biopsy, desidustat, pure red cell aplasia (prca)

## Abstract

Pure red cell aplasia (PRCA), a rare hematological disorder marked by severe anemia and reticulocytopenia, results from the near absence of developing erythroid precursors in the otherwise normal bone marrow. This case report focuses on a 48-year-old female with chronic kidney disease (CKD) who received erythropoietin injections for CKD-related secondary anemia. Despite an initial positive response, a sudden drop in hemoglobin levels prompted investigations, revealing endogenous erythropoietin (EPO)-induced PRCA due to anti-EPO antibodies. In response, desidustat, an oral hypoxia-inducible factor-prolyl hydroxylase inhibitor, was successfully introduced as an alternative treatment. This led to a substantial and sustained improvement in hemoglobin levels, emphasizing the crucial role of swift diagnosis and intervention in EPO-induced PRCA cases. Administration method and storage conditions are noteworthy factors influencing recombinant human erythropoietin (rHuEPO) immunogenicity. The case underscores desidustat's emergence as a less immunogenic and effective alternative for anemia, marking a significant advancement, particularly in the context of this pioneering case in India showcasing its efficacy.

## Introduction

Pure red cell aplasia (PRCA) is a rare hematological disorder characterized by severe anemia and reticulocytopenia (<10 x 10^9^/L) due to the nearly complete absence of developing erythroid precursors in morphologically normal bone marrow [1]. PRCA is seen in individuals with chronic kidney disease (CKD) undergoing treatment with erythropoiesis-stimulating agents (ESAs). This reaction is triggered by antibodies, which neutralize administered and endogenous erythropoietin (EPO), halting bone marrow maturation at the early erythroid progenitor stage. While many cases of acquired PRCA are idiopathic, others can be linked to underlying conditions such as thymoma, myelodysplastic syndromes, lymphoma, leukemia, systemic autoimmune disorders, and viral infections (e.g., parvovirus B19). Drug-induced PRCA may also result from treatments with medications such as phenytoin or chloramphenicol [2-4].

Most reported cases involve patients receiving epoetin therapy for anemia associated with CKD, particularly with a specific epoetin alfa product. The altered antigenicity of this product has been proposed as the underlying cause for the development of anti-EPO antibodies. Almost all documented cases of PRCA mediated by anti-EPO antibodies have been identified in CKD patients who received the drug subcutaneously [5]. EPO-related PRCA cases were mainly seen in kidney failure patients across Europe, the UK, Canada, Australia, and Asia. Despite the widespread use of epoetin and other ESAs, the reported incidence from 1989 to June 2004 was 1.6 per 10,000 patient-years of subcutaneous exposure [6-8]. In India, cases of PRCA linked to recombinant human erythropoietin (rHuEpo) are underreported, likely due to limited awareness and diagnostic accessibility, despite prolonged use for CKD-associated anemia. Until 2019, only a few rHuEpo-related cases were documented [9].

This case report highlights an instance of PRCA secondary to epoetin in an elderly female and outlines its treatment with besidustat, an oral hypoxia-inducible factor-prolyl hydroxylase inhibitor (HIF-PHI). Desidustat has demonstrated efficacy against EPO-induced PRCA by regulating EPO expression and promoting erythropoiesis [10]. The article underscores the importance of early diagnosis and treatment of PRCA in this elderly female patient, likely representing the first case in India to demonstrate the efficacy of desidustat and its benefits over EPO.

## Case presentation

A 48-year-old female was diagnosed with CKD three years ago, with bilateral small kidneys and proliferative lupus nephritis as the underlying cause. Her baseline creatinine was 1.9 mg/dL. In June 2022, her hemoglobin (Hb) concentration dropped to 7.0 gm/dL, leading to a diagnosis of secondary anemia due to CKD. Subsequently, she began receiving subcutaneous injections of rHuEpo, specifically epoetin-alfa, at a dose of 4,000 IU once a week. Her anemia responded well to this treatment, and her Hb level increased to 8.9 gm/dL over six months. While her serum creatinine level had risen to 4.38 mg/dL during this time, she continued with conservative treatment. Unexpectedly, her Hb levels dropped to 5.2 g/dL in March 2023 without any signs of bleeding, hemolysis, or infection. In response, the rHuEPO dosage was escalated to 10,000 units once a week, but there was minimal improvement, lasting only a month. After that, her Hb levels declined rapidly, falling below 4.0 gm/dL (Table 1).

**Table 1:** Changes in laboratory values during the clinical course Hb: Hemoglobin; TIBC: Total Iron-Binding Capacity; TSAT: Transferrin Saturation; LDH: Lactate Dehydrogenase

Investigation	June 2020	June 2022	Dec. 2022	March 2023	April 2023	May 2023	June 2023	September 2023	Reference range
Creatinine (mg/dL)	1.9	2.9	4.38	4.22	4.57	4.69	4.72	4.91	0.66-1.25 mg/dL
Hb (gm/dL)	11.9	7.0	8.9	5.2	6.7	4.0	3.7	9.0	11-14 gm/dL
Iron (µg/dL)	-	-	-	-	-	-	146.22	-	60-165 µg/dL
TIBC (µg/dL)	-	-	-	-	-	-	251.98	-	250-400 µg/dL
TSAT (%)	-	-	-	-	-	-	58.02%	-	30-50%
FERRITIN (ng/mL)	-	-	-	-	-	-	1255	-	4.63-200 ng/mL
LDH (U/L)	-	-	-	-	-	-	197	-	120-246 U/L
Reticulocyte count	-	-	-	-	-	-	4.5 x 10^9^/L	-	(4-5)x 10^9^/L
Parvo virus B19 DNA	-	-	-	-	-	-	Not Detected	-	-

In June 2023, a routine blood test indicated a Hb concentration of 3.7 gm/dL, prompting an investigation into the cause of anemia. Laboratory results revealed a normal iron profile. Parvovirus B19 DNA was not detected. Her liver function tests were also normal. A bone marrow biopsy revealed significant underdevelopment in the erythroid line, accompanied by overgrowth in the granulocytic and megakaryocytic lines. The ratio of blast cells was within the expected range, and there was no observable morphologic dysplasia. This clinical manifestation raised concerns about erythropoiesis-stimulating agent (ESA)-induced PRCA. In response, desidustat was initiated thrice weekly to replace the ESAs. Subsequent assessments revealed a significant change in Hb concentration.

Importantly, this case did not reveal other potential factors contributing to pure red cell aplasia (PRCA), including thymoma, hemolysis, lymphoproliferative disorders, infection, or severe malnutrition. There were no extra-renal manifestations of systemic lupus erythematosus, and complement levels were normal, indicating a quiescent effect of systemic lupus erythematosus. It is important to note that neither the patient nor her relatives made any errors in storing and handling rHuEPO. Specifically, there was no deviation from the recommended cold chain procedures. Unfortunately, we did not perform an anti-EPO antibody enzyme-linked immunosorbent assay (ELISA) as the patient could not afford it. Hence, this instance can be classified as a suspected case with bone marrow confirmation of PRCA, aligning with proposed case definitions for suspected or proven EPO-induced PRCA in individuals undergoing epoetin treatment [1]. Following treatment with desidustat at a dosage of 100 mg, administered thrice a week, her Hb levels consistently improved, reaching and maintaining levels above 9 gm/dL. We opted not to repeat the bone marrow study because of evident clinical indications of stable recovery. The data presented above suggest that the prompt diagnosis and initiation of desidustat were successful in effectively treating PRCA for this patient.

## Discussion

According to Casadevall et al., rHuEPO-associated PRCA is a rare clinical syndrome defined by three criteria: resistance to EPO therapy; anemia accompanied by reticulocytopenia (<10 × 10^9^/L) during rHuEPO therapy, either with or without the need for transfusions due to erythroblastopenia in morphologically normal bone marrow; and the presence of neutralizing anti-EPO antibodies [1]. Due to financial constraints, anti-EPO antibody testing was deemed unnecessary for our patient, meeting the first two criteria. The pathophysiologic process associated with rHuEPO-induced PRCA is complex. According to Macdougall et al., a breach in immunological tolerance triggers autoreactive T cells to recognize antigens more frequently. This leads to a B-cell-mediated antibody response in genetically sensitive individuals (HLA-DRB1*9 positive) [11]. Various factors, including advanced age, concurrent infections, intermittent illness, adjuvant therapies, comorbidities, and immune status, may collectively contribute to immune dysregulation. Anti-EPO antibodies can prevent EPO from attaching to its receptor, thus hindering erythroid progenitor differentiation [12].

The mode of treatment may also influence the immunogenicity of rHuEPO. Earlier research suggests a higher incidence of PRCA after subcutaneous administration than intravenous administration. The subcutaneous method of administration, attributed to a slower absorption rate, might result in increased recognition of antigens and presentation by cutaneous Langerhans cells. Moreover, self-administration is facilitated by the subcutaneous route, potentially leading to inadequate handling at home [9]. The use of an EPO stabilizer and how rHuEPO is stored could be another factor influencing its immunogenicity. rHuEPO is susceptible to increased temperature during storage and transport. Previously, human serum albumin was utilized for its low immunogenicity and protein-stabilizing effects. However, recent formulations incorporating polysorbate 80 and tungsten (found in vials) induce conformational changes in protein moieties, leading to increased aggregation and antigenicity. Denaturation caused by a cold chain failure may be a critical inducer of antibody production related to the EPO stabilizer polysorbate-80 (PS-80). Studies have demonstrated that PS-80 can enhance drug immunogenicity and protein aggregation [13-15].

The immunogenicity of rHuEPO may also be influenced by exposing its concealed epitopes in different packaging, storage, and transportation environments. Healthcare practitioners should be instructed to store rHuEPO between 2 and 8 °C in clinical settings and avoid breaking the cold chain or exposing the medication to shock, light, or freezing temperatures. After reformulated formulations of epoetin-α were used subcutaneously outside of the United States, a rise in cases of rHuEPO-associated PRCA occurred in the mid-1990s until 2004-2005 [16]. Of the five reported cases of rHuEPO-associated PRCA in India, four exhibited heightened levels of anti-EPO antibodies, as evidenced by a radio immunolabeled precipitation assay. Two were administered Eprex, and one was given Wepox (a follow-on epoetin-α). Comprehensive follow-up information was accessible for four cases. Two of these cases demonstrated successful hematological recovery following immunosuppressive therapy and renal transplantation; one exhibited a notable response to rituximab therapy, while another individual achieved recovery after being rechallenged with a different rEPO (darbepoetin-α) in conjunction with immunosuppressive therapy [9]. A bone marrow biopsy is the investigation of choice for diagnosing EPO-induced PRCA. The aplasia often manifests seven to eight weeks following erythropoietin treatment, attributed to the approximately 120-day lifespan of the erythrocytes generated prior to delivery. The erythropoietin-damaged bone marrow would no longer be able to manufacture new erythrocytes following their breakdown in the spleen, and anemia symptoms would manifest [17].

Desidustat is a new drug for the treatment of anemia. Individuals with kidney disease retain the ability to generate EPO, and desidustat, categorized as an oral HIF-PHI, triggers the production of endogenous EPO, maintaining levels within or close to the physiological range and resulting in an elevation of Hb levels. It is less immunogenic than rHuEPO and avoids stimulating the subcutaneous immune system [10,18]. Desidustat, possessing an utterly distinct chemical composition from rHuEPO, induces endogenous EPO at considerably lower peaks than conventional rHuEPO [10]. In our patient, treatment with desidustat was successful, increasing and maintaining the patient's Hb levels. This case report is likely the first in India to demonstrate the efficacy of desidustat and highlight its advantages over traditional EPO.

## Conclusions

Management of patients with anemia in chronic kidney disease, especially those unable to maintain Hb levels with subcutaneous injections of high-dose rHuEPO, requires careful consideration. The potential occurrence of anti-EPO antibody-mediated PRCA adds a critical dimension to the clinical landscape. This case report sheds light on early diagnosis and the efficacy of desidustat in managing such patients.
